# Improved clinical outcomes in response to a 12-week blended digital and community-based long-COVID-19 rehabilitation programme

**DOI:** 10.3389/fmed.2023.1149922

**Published:** 2023-05-24

**Authors:** Jemma L. Smith, Kevin Deighton, Aidan Q. Innes, Marc Holl, Laura Mould, Zhining Liao, Patrick Doherty, Greg Whyte, James A. King, Davina Deniszczyc, Benjamin M. Kelly

**Affiliations:** ^1^Nuffield Health, Epsom, United Kingdom; ^2^Department of Health Sciences, University of York, York, United Kingdom; ^3^School of Sport and Exercise Sciences, Liverpool John Moores University, Liverpool, United Kingdom; ^4^National Centre for Sport and Exercise Medicine, Loughborough University, Loughborough, United Kingdom; ^5^NIHR Leicester Biomedical Research Centre, University Hospitals of Leicester NHS Trust, University of Leicester, Leicester, United Kingdom; ^6^Department of Health Professions, Faculty of Health and Education, Manchester Metropolitan University, Manchester, United Kingdom

**Keywords:** coronavirus, SARS-CoV-2, exercise, emotional wellbeing, digital health, fatigue, SeaCole

## Abstract

**Introduction:**

Two million people in the UK are experiencing long COVID (LC), which necessitates effective and scalable interventions to manage this condition. This study provides the first results from a scalable rehabilitation programme for participants presenting with LC.

**Methods:**

601 adult participants with symptoms of LC completed the Nuffield Health COVID-19 Rehabilitation Programme between February 2021 and March 2022 and provided written informed consent for the inclusion of outcomes data in external publications. The 12-week programme included three exercise sessions per week consisting of aerobic and strength-based exercises, and stability and mobility activities. The first 6 weeks of the programme were conducted remotely, whereas the second 6 weeks incorporated face-to-face rehabilitation sessions in a community setting. A weekly telephone call with a rehabilitation specialist was also provided to support queries and advise on exercise selection, symptom management and emotional wellbeing.

**Results:**

The 12-week rehabilitation programme significantly improved Dyspnea-12 (D-12), Duke Activity Status Index (DASI), World Health Orginaisation-5 (WHO-5) and EQ-5D-5L utility scores (all *p* < 0.001), with the 95% confidence intervals (CI) for the improvement in each of these outcomes exceeding the minimum clinically important difference (MCID) for each measure (mean change [CI]: D-12: −3.4 [−3.9, −2.9]; DASI: 9.2 [8.2, 10.1]; WHO-5: 20.3 [18.6, 22.0]; EQ-5D-5L utility: 0.11 [0.10, 0.13]). Significant improvements exceeding the MCID were also observed for sit-to-stand test results (4.1 [3.5, 4.6]). On completion of the rehabilitation programme, participants also reported significantly fewer GP consultations (*p* < 0.001), sick days (*p* = 0.003) and outpatient visits (*p* = 0.007) during the previous 3 months compared with baseline.

**Discussion:**

The blended and community design of this rehabilitation model makes it scalable and meets the urgent need for an effective intervention to support patients experiencing LC. This rehabilitation model is well placed to support the NHS (and other healthcare systems worldwide) in its aim of controlling the impacts of COVID-19 and delivering on its long-term plan.

**Clinical trial registration:**

https://www.isrctn.com/ISRCTN14707226, identifier 14707226.

## Introduction

COVID-19 is a highly infectious respiratory disease that has elicited catastrophic health, care and economic effects. Officially declared as a global pandemic by the World Health Organization (WHO) in March 2020, there have since been 523 million cases of COVID-19, of which six million cases have resulted in death (as of May 2022) (1). Though the majority of patients recover from acute COVID-19 infection, it is now evident that survivors are at risk of suffering further long-term adverse side effects (2). Prolonged complications of COVID-19, or “long-COVID,” is defined by the National Institute for Health and Care Excellence (NICE) as “a set of persistent physical, cognitive and/or psychological symptoms that continue for more than 12 weeks after illness and which are not explained by an alternative diagnosis” (3).

The most frequently reported symptoms of long COVID (LC) include breathlessness and fatigue, as well as impaired pulmonary function and reductions in health-related quality of life (HR-QoL) (4, 5). In May 2022, it was estimated that two million people in the UK were experiencing LC (6), of which 1.4 million people reported that their day-to-day activities were adversely effected. While some symptoms of LC ease over time, research demonstrates that certain symptoms such as dyspnea and weakness are repeatedly evident up to a year post-infection. The risk for lingering LC symptoms is higher in adults aged 41–60 and >60 years as well as in those who were unvaccinated against COVID-19 (7). To date, evidence-based guidelines do not exist for the treatment of LC and its associated complications, with NICE highlighting the need for effective interventions to manage this condition (3). Outpatient pulmonary rehabilitation, a multi-component exercise and education programme for patients with chronic lung disease, has now been shown effective in improving the symptoms of LC (2). However, a scalable solution, enrolled via a community rehabilitation setting, is required due to the large number of people experiencing this condition and to reduce strain on an already pressured healthcare system.

The purpose of this study was to assess the clinical effectiveness of a novel 12-week blended community rehabilitation programme for individuals experiencing LC. These are the first results from a scalable rehabilitation programme for participants presenting with LC. The findings demonstrate substantial improvements in LC symptoms and suggest that this rehabilitation model can support the NHS (and other healthcare systems worldwide) in its aim of controlling the impacts of COVID-19 and delivering on its long-term plan.

## Materials and methods

### Study design and participants

This study was conducted according to the guidelines laid down in the Declaration of Helsinki, and all procedures were approved by the Ethics Advisory Committee at Manchester Metropolitan University (Ref: 25307). Participants were able to enroll into the rehabilitation programme online via self-referral or referral from an NHS practitioner. Following enrollment, participants underwent triage with a physiotherapist to assess eligibility for inclusion in the study. Eligible participants were then assigned a start date for the programme.

All participants reported in this manuscript provided written informed consent for the inclusion of outcomes data in external publications.

This service evaluation used baseline and follow-up data (at 12 weeks) from 601 people with LC undertaking the Nuffield Health COVID-19 Rehabilitation Programme between February 2021 and March 2022. This trial is registered at the ISRCTN Registry (ID: 14707226). A full overview of inclusion and exclusion criteria is provided in Table 1.

**TABLE 1:** Study inclusion and exclusion criteria.

Inclusion criteria	Exclusion criteria
Previous diagnosis of COVID-19	Active COVID-19 symptoms
Able to walk independently for a minimum of 20 m	Are already receiving community rehabilitation
Must have access to the internet and smartphone/tablet/personal computer (with adequate technological literacy)	Have un-managed medical conditions that contraindicate unsupervised exercise
18 years of age and over	Have a formal diagnosis of post-traumatic stress syndrome, clinically significant anxiety or depression where low intensity mental health intervention will not assist
Access to transport for phase 2 attendance	Have been diagnosed with chronic fatigue syndrome prior to contracting COVID-19

### Rehabilitation programme

The LC rehabilitation programme aims to improve symptoms of LC, functional capacity, personal well-being and HR-QoL. The LC rehabilitation programme is a 12-week, group-based programme split into two 6-week phases consisting of 3 × 45-min exercise sessions per week and continued support from a programme lead, with a maximum of 10 participants per group. Table 2 details the intervention according to the Template for Intervention Description and Replication checklist.

**TABLE 2:** Overview of rehabilitation programme according to the TIDieR checklist.

Item no.	Item
**Brief name**	
1	Long-COVID rehabilitation programme
**Why**	
2	The rehabilitation programme aims to improve symptoms of Long-COVID, functional capacity, personal well-being and HR-QoL
**What**	
3	Participants had access to a web-based LC rehabilitation hub (available here: https://www.nuffieldhealth.com/covid-rehab) which contained the on-demand exercise sessions, webinars, and resources relevant to the programme. Participants were provided with a physical copy of the Nuffield Health LC rehabilitation journal, which included information, advice, and activities to support participants recovery. Participants utilized the journal to log progress by recording their goals, exercise, and general activity.
4	Over 12 weeks, participants engaged in 3 × 45-min exercise sessions per week which included a group session, a pre-recorded session, and a self-directed session. The programme was conducted remotely for the first 6 weeks and in Nuffield Health Fitness and Wellbeing Centres in the last 6 weeks. Participants also had a weekly telephone call with their rehabilitation specialist which provided support with exercise selection, symptom management, and emotional wellbeing.
**Who provided**	
5	All sessions were delivered by Nuffield Health Rehabilitation Specialists (personal trainers who had received specialist training in LC exercise modalities for LC, and methods of effective data collection). On successful completion of the LC course, the Rehabilitation Specialist has access to an online platform containing relevant materials for delivering the programme. This ensures that the delivery of the LC remains consistent in Nuffield Health Fitness and Wellbeing Centres across the UK.
**How**	
6	The LC rehabilitation programme is a group-based programme, which is split into 6 weeks of virtual provision and 6 weeks face-to-face, with a maximum of 10 participants permitted per group.
**Where**	
7	The LC programme was conducted at 51 Nuffield Health Fitness and Wellbeing Centres, all of which were registered with the Care Quality Commission (England) or the Care Inspectorate (Scotland). Nuffield Health Fitness and Wellbeing centres are commercial gyms, available to the public. The LC rehabilitation programme was free to participants.
**When and how much**	
8	The LC programme consisted of 3 × 45-min exercise sessions per week (36 in total). An overview of the components included in the programme is available in Table 3. Activity sessions included a combination of cardiovascular, strength-based and mobility exercises.
**Tailoring**	
9	Target exercise intensity and volume, as well as movement complexity, range of motion, and stability were prescribed according to the participants functional capacity and physical fitness which was recorded at baseline (using the Duke Activity Status Index and Sit-to-Stand test). See Supplementary material for more information.
**Modifications**	
10	The LC rehabilitation programme was conducted virtually for the first 6-weeks and face-to-face for the remaining 6 weeks.
**How well**	
11	Adherence to the rehabilitation programme was logged by the rehabilitation specialist leading the session manually.

Throughout the 12-week programme participants completed three exercise sessions per week: (1) group rehabilitation exercise session lasting 45 min; (2) on-demand exercise session lasting 45 min, using a pre-recorded guided exercise session located on a dedicated online platform (Vimeo, New York, USA); (3) a “build your own” exercise session whereby participants selected from a menu of activities provided within their rehabilitation workbook to populate a session commensurate with their personal threshold. An overview of the components included in the 12-week programme are presented in Table 3.

**TABLE 3:** Components of LC rehabilitation programme.

Weeks 1–6 (virtual)	Weeks 7–12 (face-to-face)
**Weekly one-to-one call: with programme lead to guide the participant through the programme and monitor progress**		
Group live-stream exercise class: a weekly live online exercise class, led by the programme lead and attended by the participants in the same cohort.	Group exercise class: weekly group class at local Nuffield Health fitness and wellbeing centre.
**On-demand workout: exercises specifically developed for rehabilitation and covering a range of levels, available for the participant to complete in their own time, accessible here: www.nuffieldhealth.com/covid-rehab**		
“Build your own” self-directed activity: Exercise session developed by the participant and completed at home, focusing on areas relevant to recovery.	“Build your own” self-directed activity: exercise session conducted at local Nuffield Health fitness and wellbeing centre with the program lead available to provide guidance and support.

The first phase of the programme was conducted remotely in Weeks 1–6. The group exercise session was performed via an online live-streaming platform (Microsoft Teams^®^), with the programme lead delivering the session.

This session was immediately followed by a 15-min period for further questions using either the online chat function or device microphones. In accordance with the remote nature of the first phase of the rehabilitation programme, participants completed the on-demand exercise session and the “build your own” exercise session from their own homes.

The second phase of the programme was conducted in Weeks 7–12 and incorporated face-to-face rehabilitation sessions. This was achieved by conducting the group rehabilitation exercise sessions on-site at the respective Nuffield Health Fitness and Wellbeing Centre. Although the structure of the sessions remained the same as during Phase 1 of the programme, the face-to-face nature enabled two-way interaction with the rehabilitation specialist leading the session, as well as with fellow participants to foster a supportive environment. The on-demand exercise session remained as a remote aspect of the programme throughout Weeks 7–12 of the programme. Participants were encouraged to complete their “build your own” rehabilitation session within a supervised gym environment, with the rehabilitation specialist available to offer advice and guidance.

All activity sessions included a combination of cardiovascular, strength-based, and mobility exercises. Exercise intensity and volume, as well as movement complexity, range of motion and stability were prescribed according to the participants functional capacity and physical fitness, which was recorded at baseline (see Supplementary material for further information). Following exercise sessions, participants were advised to record their perceived effort score on a scale which ranged from 0 (no effort) to 10 (extremely hard). Self-recording exercise effort allowed for participants to monitor their progress and adjust effort accordingly over the 12-week programme. Participants were also encouraged to progress movement complexity, range of motion, stability, and volume where possible.

Participants also received a weekly telephone call with a rehabilitation specialist lasting up to 45 min to support with any queries on the programme and provide advice on exercise selection, symptom management and emotional wellbeing. Participants had access to a web-based LC rehabilitation hub^1^ which contained the on-demand exercise sessions, webinars, and resources relevant to the programme. Participants were provided with a printed copy of the Nuffield Health LC rehabilitation journal which contained information on physical and emotional wellbeing, and an activity record to log exercise sessions completed each week.

The LC programme was conducted at 51 Nuffield Health Fitness and Wellbeing Centres, all of which were registered with the Care Quality Commission (England) or the Care Inspectorate (Scotland). Nuffield Health Fitness and Wellbeing centres are commercial gyms, available to the public. The LC rehabilitation programme was free to participants.

Full details of the rehabilitation programme are provided in the published study protocol (8).

### Training of rehabilitation specialists

All sessions were delivered by Nuffield Health Rehabilitation Specialists (personal trainers who had received specialist training in LC exercise modalities for LC, and methods of effective data collection). On successful completion of the LC course, the Rehabilitation Specialist has access to an online platform containing relevant materials for delivering the programme. This ensures that the delivery of the LC remains consistent in Nuffield Health Fitness and Wellbeing Centres across the UK.

#### Measures

Outcome data were collected for all participants at baseline and on completion of the rehabilitation programme at Week 12 via a digital application (MyWellbeing, Nuffield Health, London, UK). Data were objectively measured by the Rehabilitation Specialist or self-reported and stored on a web-based platform (Lumeon, London, UK).

#### Dyspnea

Breathlessness was measured using the Dyspnea-12 tool (D-12) (9). The D-12 assesses multiple breathless sensations within a single instrument. Total scores from the D-12 range from 0 to 36, with higher scores corresponding to greater severity of breathlessness.

#### Functional capacity

Functional capacity was assessed using the Duke Activity Status Index (DASI) (10). The DASI produces a score between 0 and 58.2 points, with higher scores indicating a higher functional status.

#### Physical fitness

Physical fitness was assessed using the 30-s sit-to-stand test (11). The 30-s sit-to-stand test involves recording the number of stands a person can complete in 30 s. Higher scores indicate a greater fitness level.

#### Mental wellbeing

Mental wellbeing was assessed using The World Health Organization- Five Well-Being Index (WHO-5) (12). The WHO-5 is a short, generic global rating score which measures subjective wellbeing. The WHO-5 produces a score between 0 and 25 which is translated to a percentage scale from 0 (absence of wellbeing) to 100 (maximal wellbeing).

#### Health status

Health status was assessed using the EuroQoL Five Dimension Five Level (EQ-5D-5L) and visual analog scale (VAS) (13). The descriptive system consists of five dimensions: mobility, self-care, usual activities, pain/discomfort, and anxiety/depression. Patients indicate their health state by ticking the box next to the appropriate statement: no problems, slight problems, moderate problems, severe problems, and extreme problems. The EQ-5D-5L scores were mapped to utility values in accordance with the England-specific valuation set (14). The EQ VAS records health on a vertical VAS, where the endpoints are labeled “The best health you can imagine” for a score of 100 and “The worst health that you can imagine” for a score of 0.

#### Illness burden

At baseline and Week 12, participants also reported the number of general practitioner (GP) consultations, outpatient hospital episodes, inpatient hospital episodes and days absent from work due to illness (“sick days”) experienced during the past 3-months (for participants who were engaged in employment at baseline). This enabled a comparison of illness burden during the rehabilitation programme compared with the preceding 3-month period.

### Statistical analysis

The primary analyses were conducted in 601 participants who completed the LC rehabilitation programme and provided outcome measures at Week 0 and 12. A subgroup of 539 participants provided D-12, DASI, WHO-5, EQ-5D-5L and sit-to-stand test outcomes at the midpoint of the intervention. The outcomes for these participants were analyzed at Weeks 0, 6, and 12 to understand the time-course of changes in response to the programme.

Graphical representations of the results are provided as mean (95% confidence interval [CI]). Descriptions of data in the text for individual timepoints are provided as mean (SD), with the difference between timepoints provided as mean (95% CI). Paired samples *t*-tests were used to determine significant differences between timepoints. Additionally, the 95% CIs of differences between timepoints were compared against the minimum clinically important difference (MCID) thresholds identified for relevant outcomes from the wider research literature. Where the 95% CIs of a difference between timepoints exceeded the MCID, this demonstrated that the change was significantly greater than the MCID. Two-sided 95% CIs were used for all analyses and all significance tests were performed at the 5% alpha level.

The MCID for each outcome was obtained from the research literature, as follows: D-12 score (2.83 points) (15), WHO-5 score (10 points) (16), EQ-5D-5L utility (0.05 points) (17), EQ-5D VAS (7 points) (17), sit-to-stand score (2 points) (11). To the authors’ knowledge a MCID is not established for DASI score; consequently, a threshold of 5 points was used based on this representing a significant difference in score between patients who did versus did not suffer 1-year new disability or death after non-cardiac surgery (18).

All analyses were performed using R version 4.1.2 (19).

## Results

Complete case analysis was conducted for the 601 participants who completed the Nuffield Health COVID-19 Rehabilitation Programme and provided the relevant outcome measures at Weeks 0 and 12.

### Participant characteristics

All participants had been previously diagnosed with COVID-19 and were currently experiencing symptoms of LC, with a mean period of 9.8 (SD 5.0) months between COVID-19 diagnosis and commencing the rehabilitation programme. During COVID-19 infection 13.3% of participants were admitted to hospital, with a mean hospital stay duration of 10 (SD 16) days and 16.5% of these participants being admitted to an intensive care unit. On programme enrollment, participants had a mean age of 47 (SD 10) years and 2.9 (SD 1.7) comorbidities. The majority of participants were female (77.4%) and of White British ethnicity (88.6%). Participant characteristics are presented in Table 4.

**TABLE 4:** Participant characteristics.

	Frequency	%
Age (years)	47 (10)	
**Gender**		
Male	136	22.6
Female	465	77.4
**Ethnicity**		
African	2	0.3
Any other Asian background	1	0.2
Any other ethnic group	3	0.5
Any other mixed background	4	0.7
Any other white background	25	4.2
Caribbean	2	0.3
Chinese	1	0.2
Indian	10	1.7
Pakistani	3	0.5
White and Asian	3	0.5
White and black African	3	0.5
White and black Caribbean	5	0.8
White British	531	88.6
White Irish	6	1.0
**Employment status**		
Employed	406	67.9
In full-time education	15	2.5
Retired	21	3.5
Unable to work due to condition	137	22.9
Unemployed	19	3.2
Number of comorbidities	2.9 (1.7)	
Months since initial COVID-19 infection	9.8 (5.0)	
**Admitted to hospital following initial COVID-19 infection**		
Yes	79	13.3
No	515	86.7
Length of hospital stay (days)	10 (16)	
**Admission to intensive care unit**		
Yes	13	16.5
No	63	79.7
N/A	3	3.8

Values are presented as frequency (%) for categorical variables and mean (standard deviation) for continuous variables.

**FIGURE 1:**
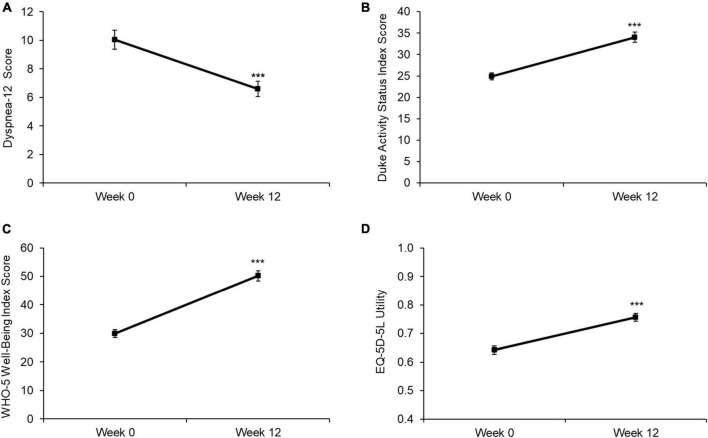
Baseline and Week 12 values for **(A)** D-12 score, **(B)** DASI score, **(C)** WHO-5 score, and **(D)** EQ-5D-5L utility. Values are presented as mean (95% CI). Higher scores indicate a worse health state for D-12, while lower scores indicate a worse health state for DASI, WHO-5, and EQ5D. Differences between time points were analyzed using paired *t*-tests. ***p < 0.001. D12 score: *n* = 598; DASI score: *n* = 594; WHO-5 score: *n* = 600; EQ5D utility: *n* = 600.

The 12-week rehabilitation programme significantly improved D-12, DASI, WHO-5 and EQ-5D-5L utility values (Figure 1; all *p* < 0.001). Additionally, the 95% CIs for the improvement in each of these outcomes exceeding the MCID for each measure (mean change [CI]: D-12: −3.4 [−3.9, −2.9]; DASI: 9.2 [8.2, 10.1]; WHO-5: 20.3 [18.6, 22.0]; EQ-5D-5L utility: 0.11 [0.10, 0.13]). Significant improvements in EQ-5D VAS (Week 0: 47.5 [20.6]; Week 12: 62.2 [23.6]; *p* < 0.001) and sit-to-stand test results (Week 0: 11.5 [4.8]; Week 12: 15.6 [6.1]; *p* < 0.001) were also observed, with the 95% Cis of the improvements exceeding the MCID for each measure (EQ5D VAS: 14.7 [12.6, 16.9]; sit-to-stand test: 4.1 [3.5, 4.6]).

**FIGURE 2:**
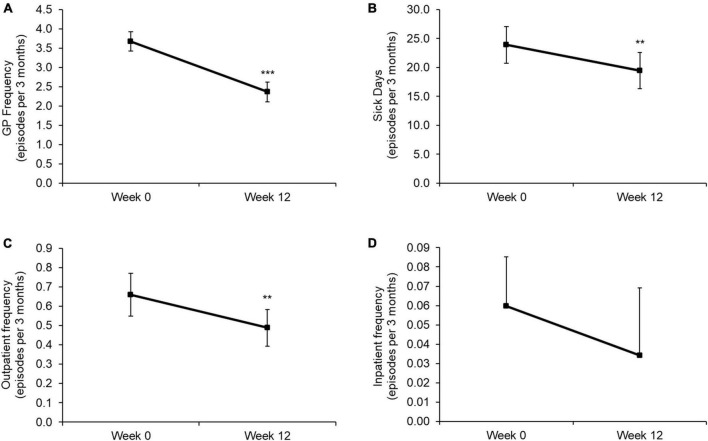
Baseline and Week 12 values for **(A)** the number of GP consultations, **(B)** sick days, **(C)** outpatient admissions, and **(D)** inpatient admissions during the past 3 months. Values are presented as mean (95% CI). Differences between time points were analyzed using paired *t*-tests. **p < 0.01; ***p < 0.001. GP frequency: *n* = 585; sick days: *n* = 402; outpatient frequency: *n* = 585; inpatient frequency: *n* = 585. Note, the lower error bar has been removed from panel **(D)** due to this crossing the *x*-axis.

On completion of the rehabilitation programme, participants reported significantly fewer GP consultations (Figure 2A; *p* < 0.001), sick days (Figure 2B; *p* = 0.003) and outpatient visits (Figure 2C; *p* = 0.007) during the previous 3 months compared with baseline. The number of inpatient episodes experienced during the past 3 months did not change in response to the rehabilitation programme but was limited by the small number of episodes at baseline (Figure 2D; *p* = 0.235).

**FIGURE 3:**
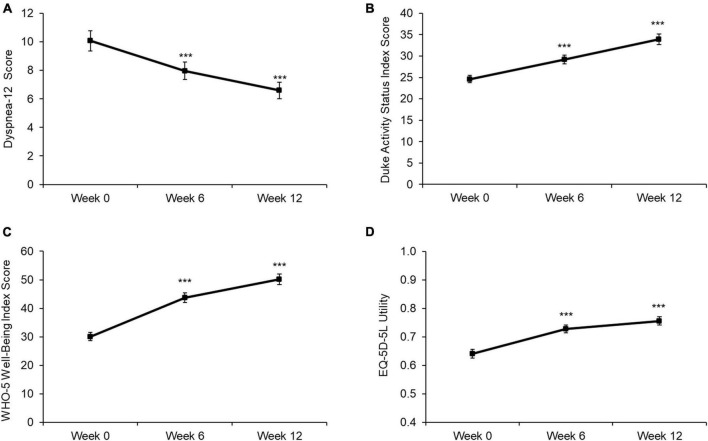
Baseline, Week 6, and Week 12 values for **(A)** D-12 score, **(B)** DASI score, **(C)** WHO-5 score, and **(D)** EQ-5D-5L utility. Values are presented as mean (95% CI). Higher scores indicate a worse health state for D-12, while lower scores indicate a worse health state for DASI, WHO-5, and EQ5D. Differences between time points were analyzed using paired *t*-tests. ***p < 0.001. D12 score: *n* = 535; DASI score: *n* = 528; WHO-5 score: *n* = 538; EQ5D utility: *n* = 526.

A subgroup of patients with measures collected at Weeks 0, 6, and 12 demonstrated significant improvements in D-12, DASI, WHO-5 and EQ-5D-5L utility values from Week 0 to 6 (all *p* < 0.001), and further significant improvements from Week 6 to 12 (all *p* < 0.001) (Figure 3). Significant improvements for Week 0–6 and Week 6–12 were also observed for EQ-5D VAS (Week 0: 47.2 [20.7]; Week 6: 55.8 [25.0]; Week 12: 62.0 [23.9]; both *p* < 0.001) and sit-to-stand test results (Week 0: 11.5 [4.2]; Week 6: 13.9 [4.5]; Week 12: 15.4 [5.3]; both *p* < 0.001).

Changes in the individual components of the D-12, DASI, WHO-5 and EQ-5D-5L questionnaires are available in the Supplementary material for the primary analysis (Week 0 to 12) and time-course analysis (Week 0 to 6 to 12).

## Discussion

This is the first evaluation of the effectiveness of a scalable community rehabilitation programme for participants presenting with LC. The outcomes demonstrate significant and clinically meaningful improvements in dyspnea, functional capacity, wellbeing and HR-QoL at programme completion. A reduced number of GP consultations, sick days and outpatient admissions were also observed during the programme compared with the preceding 3-month period. These findings suggest that the Nuffield Health COVID-19 Rehabilitation Programme can meet the urgent need for an effective and scalable LC rehabilitation model.

The improvements in functional capacity in response to the rehabilitation programme were demonstrated via a mean 9.2 point (37%) improvement in DASI score and a 4.1 score (36%) improvement in the 30-s sit-to-stand test. While the precise mechanisms of LC remain largely unknown (20), these findings suggest that the underlying pathology is responsive to exercise. The improvements in functional capacity are also likely to be linked with the observed reduction in dyspnea, as demonstrated through a mean 3.4 point (34%) improvement in D-12 score. Though several studies have utilized the 6-min walk test distance to assess improvements in exercise tolerance following pulmonary rehabilitation for LC (21), the sit-to-stand test is also highly recommended in the LC population (22). The sit-to-stand test was selected as it is simple to set up, easily conducted by trained, non-medical personnel, and manageable in restricted spaces. The exact mechanisms underlying exercise-induced LC improvements are yet to be fully elucidated, however, exercise is known to elicit structural and functional adaptations of the cardiovascular and musculoskeletal systems including enhanced lung function and respiratory muscle strength (23). Indeed, previous research in COPD patients has consistently demonstrated beneficial changes in exertional ventilation, breathing pattern, operating lung volume and static respiratory muscle strength in response to exercise training (24), even despite variability in the nature and composition of the rehabilitation protocols.

Consistent with the improvements in physical symptoms, significant and clinically meaningful increases in mental wellbeing and HR-QoL were observed in response to the 12-week rehabilitation programme. Participants demonstrated a mean 20.3 point (68%) improvement in WHO-5 score as a marker of mental wellbeing, alongside marked improvements in EQ-5D-5L utility and VAS scores. The mental wellbeing benefits of exercise are well documented in various chronic diseases including cardiovascular disease, diabetes and obesity, with regular physical activity shown to improve overall mood and symptoms of anxiety and depression (25). These effects may have been enhanced by the programme design of group exercise sessions and regular interactions with a rehabilitation specialist to increase feelings of social support and social connectedness with LC sufferers. These findings are notable, especially when considering that symptoms of PTSD, anxiety, and depression are frequently reported after COVID-19 infection, regardless of hospitalization status (26). As such, multidisciplinary rehabilitation models, targeting both the physical and mental health symptoms of LC are of critical importance, with the observed improvements in HR-QoL likely to be the result of combined improvements in both mental and physical wellbeing (27).

The improvements in physical and mental health and HR-QoL appeared to translate into reduced healthcare utilization during the rehabilitation programme. In this regard, participants reported significantly fewer GP consultations, sick days, and outpatient admissions during the 12-week rehabilitation programme compared with the 3-month period preceding the programme. This aligns with previous research in COPD patients showing reduced hospital readmission rates and medical costs during the 6 months after completion of a pulmonary rehabilitation programme, compared with patients who did not initiate rehabilitation (28). It is important to note that recall bias may have influenced the accuracy of participants reporting of healthcare utilization over the past 3 months. Nevertheless, considering the economic consequences of sickness absence and health-related productivity losses (29), as well as the healthcare resource utilization required for the treatment of LC, further investigation into the benefits of LC rehabilitation on these outcomes may be beneficial to understand the wider societal impacts of this intervention.

Importantly, beneficial effects of the rehabilitation programme for physical and mental health and HR-QoL were observed after 6 weeks, with further improvements then observed at 12 weeks. This finding strengthens the theory that pulmonary rehabilitation programs for LC may benefit from a duration of 12 weeks, rather than the 6–8 week duration seen in some studies (30). During the first 6 weeks of the programme all activities were conducted remotely, which demonstrates the effectiveness of a telerehabilitation model for LC rehabilitation. Further research is required to determine whether the additional improvements from 6 to 12 weeks occurred as a result of the extended duration or the incorporation of face-to-face rehabilitation sessions. Previous research in COPD has demonstrated that remote delivery of pulmonary rehabilitation provides similar outcomes to face-to-face PR, with improvements in exercise capacity, dyspnea and HR-QoL (31). These findings suggest that a fully remote or blended rehabilitation programme provides a clinically effective alternative to centre-based approaches for LC, with a fully remote model representing a potential approach to increase the reach of the programme while maintaining effectiveness.

### Limitations

The present study has demonstrated the clinical effectiveness of a scalable LC rehabilitation model for improving physical, mental, and HR-QoL outcomes. This is particularly striking considering current evidence that LC does not appear to improve over time in the absence of targeted therapies (32). Nevertheless, some limitations must be acknowledged. First, a control group was not included. Therefore, we cannot directly attribute all of the observed benefits to the LC rehabilitation programme or understand the influence of factors such as support from Rehabilitation Specialists or social interaction with other participants. Nonetheless, the observed benefits exceed any improvements reported without intervention in the literature. Second, the majority of patients were female (77.4%) and of White British ethnicity (88.6%). Although LC is more common in women than men (33), the lack of diversity in this sample demonstrates the need for further work to engage with additional populations and communities. Further, people who were previously diagnosed with chronic fatigue syndrome prior to COVID-19 infection were excluded from the study, therefore the findings cannot be extended to this population. Third, we did not utilize a population-specific functionality measure such as the post-COVID functional status scale (PCFS) (34). This could be utilized in future research to monitor changes in function. Last, the effectiveness of the rehabilitation programme was monitored over a relatively short period of 12 weeks. Research in COPD suggests that the benefits of PR may last for 4 years after program discharge; however, further data maturity is required to understand whether the benefits observed in response to the present programme remain after completion.

## Conclusion

In conclusion, this evaluation demonstrated significant and clinically meaningful improvements in dyspnea, functional capacity, mental wellbeing and HR-QoL in response to a 12-week rehabilitation programme for participants presenting with LC. A reduced number of GP consultations, sick days and outpatient admissions was also observed during the 3-month programme. The blended and community design of this rehabilitation model makes it scalable and meets the urgent need for an effective intervention to support patients experiencing LC. Improvements observed during the first 6 weeks of the programme also demonstrate the effectiveness of remote programme delivery. This rehabilitation model is well placed to support the NHS (and other healthcare systems worldwide) in its aim of controlling the impacts of COVID-19 and delivering on its long-term plan.

## Data availability statement

The original contributions presented in this study are included in the article/Supplementary material, further inquiries can be directed to the corresponding author.

## Ethics statement

The studies involving human participants were reviewed and approved by the Manchester Metropolitan University. The patients/participants provided their written informed consent to participate in this study.

## Author contributions

AI, MH, LM, ZL, PD, GW, JK, DD, and BK contributed to the design and coordination of the intervention. KD performed the statistical analysis. JS and KD drafted the manuscript. BK accepted the full responsibility for the work and the decision to publish and attested that all authors meet authorship criteria and no others meeting the criteria have been omitted. All authors contributed to the final manuscript.

## Footnotes

1
https://www.nuffieldhealth.com/covid-rehab
