# Supplementary material for: Improved clinical outcomes in response to a 12-week blended digital and community-based long-COVID-19 rehabilitation programme
Source: Front Med (Lausanne). 2023 May 24;10:1149922. doi: 10.3389/fmed.2023.1149922 (PMC10244528; doi:10.3389/fmed.2023.1149922)
Supplement: Supplementary file 1 [file Data_Sheet_1.DOCX]

**Supplementary Methods: Rehabilitation programme design**

**S1 Table. Exercise prescription grades based on Sit-to-Stand Test and Functional Capacity scores.**

|  | **Exercise grade and category** | | | |
| --- | --- | --- | --- | --- |
|  | **1 (Low)** | **2 (Low-Moderate)** | **3 (Moderate)** | **4 (High)** |
| Sit-to-Stand Test | ≤ 5 | ≤ 8 | ≤ 12 | ≥ 16 |
| Functional Capacity (METS) | < 4 | 4-5 | 5-8 | ≥ 8 |

**S2 Table. Target exercise intensity and volume for participants in each prescription grade.**

|  | **Exercise grade and category** | | | |
| --- | --- | --- | --- | --- |
|  | **1 (Low)** | **2 (Low-Moderate)** | **3 (Moderate)** | **4 (High)** |
| Target Rate of Perceived Exertion | 1 - 3 | 2 - 4 | 4 - 6 | 5 - 7 |
| Target Heart Rate (% max HR) | 40 - 50% | 50 - 60% | 60 - 80% | 70 - 90% |
| Target number of functional exercises to perform per session | 1 | 2 | 3 | 4 |

**S3 Table. Exercise prescription for participants in each grade.**

|  | **Exercise grade and category** | | | |
| --- | --- | --- | --- | --- |
|  | **1 (Low)** | **2 (Low-Moderate)** | **3 (Moderate)** | **4 (High)** |
| Movement complexity | - Single joint movements. - Single planes of movement. | - Multi-joint movements. - Single planes of movement. - Low stability demand. - Machine based / suspensions strap supported movements. | - Multi-joint movements. - Single planes of movement. - High stability demand. - Bodyweight or free-standing movements, unsupported. | - Multi-joint movements. - Multiple planes of movement. |
| Range of motion | - Within contraindication limits / pain limits. | - Full available / specific range strengthening. | - Range development / full range strengthening. | |
| Stability | - Simple, stable surface, wide base of support. | - Moving outside base of support, narrower base of support. | - Single leg stances, unstable surfaces. | - Complex challenges, enhancing reactive components to proprioception. |
| Volume | - Motor patterning skills, work to fatigue. | - Endurance, higher reps to fatigue. | - Potential load added under control. | - Return to normal programming protocols. |

**Supplementary Results: Changes in the individual components of the D-12, DASI, WHO-5 and EQ-5D-5L questionnaires.**

**S4 Table. Dyspnea-12 component scores at Weeks 0 and 12.**

|  | Week 0 | Week 12 | Δ Week 0 to 12 |
| --- | --- | --- | --- |
| I cannot get enough air | 0.8 (0.8) | 0.5 (0.7) | -0.3 (-0.3, -0.2)*** |
| I feel short of breath | 1.3 (0.8) | 0.9 (0.7) | -0.4 (-0.5, -0.3)*** |
| I have difficulty catching my breath | 1.0 (0.8) | 0.7 (0.7) | -0.3 (-0.4, -0.3)*** |
| My breath does not go all the way in | 0.9 (0.8) | 0.7 (0.7) | -0.2 (-0.3, -0.2)*** |
| My breathing is distressing | 0.6 (0.8) | 0.3 (0.6) | -0.3 (-0.3, -0.2)*** |
| My breathing is exhausting | 0.7 (0.8) | 0.5 (0.7) | -0.2 (-0.3, -0.2)*** |
| My breathing is irritating | 0.8 (0.9) | 0.5 (0.7) | -0.3 (-0.3, -0.2)*** |
| My breathing is uncomfortable | 0.8 (0.8) | 0.5 (0.7) | -0.3 (-0.3, -0.2)*** |
| My breathing makes me agitated | 0.6 (0.8) | 0.4 (0.7) | -0.2 (-0.3, -0.2)*** |
| My breathing makes me feel depressed | 0.6 (0.9) | 0.4 (0.7) | -0.2 (-0.3, -0.2)*** |
| My breathing makes me feel miserable | 0.7 (0.9) | 0.4 (0.7) | -0.3 (-0.4, -0.3)*** |
| My breathing requires more work | 1.2 (0.8) | 0.9 (0.7) | -0.4 (-0.4, -0.3)*** |

Values for each timepoint are presented as mean (SD), while delta values are presented as mean (95% CI). Higher scores indicate a worse health state. Differences between time points were analysed using paired t-tests. ***p < 0.001, **p < 0.01, *p < 0.05. N=598.

**S5 Table. Duke Activity Status Index component scores at Weeks 0 and 12.**

|  | Week 0 | Week 12 | Δ Week 0 to 12 |
| --- | --- | --- | --- |
| Can you take care of yourself (eating, dressing, bathing or using the toilet)? | No: 6 (1.0%)  Yes: 588 (99.0%) | No: 8 (1.3%)  Yes: 586 (98.7%) | No: 2 (0.3%)  Yes: -2 (-0.3%) |
| Can you walk indoors, such as around your house? | No: 1 (0.2%)  Yes: 593 (99.8%) | No: 2 (0.3%)  Yes: 592 (99.7%) | No: 1 (0.1%)  Yes: -1 (-0.1%) |
| Can you walk a street or two on level ground? | No: 34 (5.7%)  Yes: 560 (94.3%) | No: 18 (3.0%)  Yes: 576 (97.0%) | No: -16 (-2.7%)  Yes: 16 (2.7%)  * |
| Can you climb a flight of stairs or walk up a hill? | No: 69 (11.6%)  Yes: 525 (88.4%) | No: 51 (8.6%)  Yes: 543 (91.4%) | No: -18 (-3.0%)  Yes: 18 (3.0%) |
| Can you run a short distance? | No: 508 (85.5%)  Yes: 86 (14.5%) | No: 407 (68.5%)  Yes: 187 (31.5%) | No: -101 (-17.0%)  Yes: 101 (17.0%)  *** |
| Can you do light work around the house, such as dusting or washing dishes? | No: 20 (3.4%)  Yes: 574 (96.6%) | No: 13 (2.2%)  Yes: 581 (97.8%) | No: -7 (-1.2%)  Yes: 7 (1.2%) |
| Can you do moderate work around the house, such as hoovering, sweeping floors or carrying in groceries? | No: 193 (32.5%)  Yes: 401 (67.5%) | No: 87 (14.6%)  Yes: 507 (85.4%) | No: -106 (-17.9%)  Yes: 106 (17.9%)  *** |
| Can you do heavy work around the house, such as scrubbing floors, or lifting and moving heavy furniture? | No: 527 (88.7%)  Yes: 67 (11.3%) | No: 372 (62.6%)  Yes: 222 (37.4%) | No: -155 (-26.1%)  Yes: 155 (26.1%)  *** |
| Can you do gardening work, such as raking leaves, weeding or pushing a lawnmower? | No: 385 (64.8%)  Yes: 209 (35.2%) | No: 235 (39.6%)  Yes: 359 (60.4%) | No: -150 (-25.2%)  Yes: 150 (25.2%)  *** |
| Can you have sexual relations? | No: 253 (42.6%)  Yes: 341 (57.4%) | No: 146 (24.6%)  Yes: 448 (75.4%) | No: -107 (-18.0%)  Yes: 107 (18.0%)  *** |
| Can you participate in moderate recreational activities, such as golf, bowling, dancing or doubles tennis? | No: 448 (82.2%)  Yes: 106 (17.8%) | No: 339 (57.1%)  Yes: 255 (42.9%) | No: -149 (-25.1%)  Yes: 149 (25.1%)  *** |
| Can you participate in strenuous sports, such as swimming, singles tennis, football or skiing? | No: 572 (96.3%)  Yes: 22 (3.7%) | No: 475 (80.0%)  Yes: 119 (20.0%) | No: -97 (-16.3%)  Yes: 97 (16.3%)  *** |

Values are presented as count (%). Differences between time points were analysed using McNemar's chi-squared tests. ***p < 0.001, **p < 0.01, *p < 0.05. N = 594.

|  | Week 0 | Week 12 | Δ Week 0 to 12 |
| --- | --- | --- | --- |
| Mobility | 2.1 (0.9) | 1.8 (0.8) | -0.3 (-0.4, -0.3)*** |
| Self-care | 1.4 (0.7) | 1.2 (0.5) | -0.2 (-0.2, -0.1)*** |
| Usual activities | 3.2 (1.0) | 2.4 (1.0) | -0.8 (-0.9, -0.7)*** |
| Pain / discomfort | 2.6 (0.9) | 2.2 (0.9) | -0.4 (-0.5, -0.4)*** |
| Anxiety / depression | 2.4 (0.9) | 1.9 (0.8) | -0.5 (-0.5, -0.4)*** |

**S6 Table.** **EQ-5D-5L component scores at Weeks 0 and 12.**

Values for each timepoint are presented as mean (SD), while delta values are presented as mean (95% CI). Higher scores indicate a worse health state. Differences between time points were analysed using paired t-tests. ***p < 0.001, **p < 0.01, *p < 0.05. N = 600.

**S7 Table.** **World Health Organisation - Five Well-Being Index component scores at Weeks 0 and 12.**

|  | Week 0 | Week 12 | Δ Week 0 to 12 |
| --- | --- | --- | --- |
| I have felt cheerful and in good spirits | 2.1 (1.2) | 3.0 (1.1) | 0.9 (0.8, 1.0)*** |
| I have felt calm and relaxed | 2.1 (1.2) | 3.0 (1.2) | 0.9 (0.8, 1.0)*** |
| I have felt active and vigorous | 0.7 (0.9) | 2.0 (1.4) | 1.3 (1.1, 1.4)*** |
| I woke up feeling fresh and rested | 0.8 (1.1) | 1.8 (1.4) | 1.0 (0.9, 1.1)*** |
| My daily life has been filled with things that interest me | 1.7 (1.2) | 2.7 (1.3) | 1.0 (0.9, 1.1)*** |

Values for each timepoint are presented as mean (SD), while delta values are presented as mean (95% CI). Lower scores indicate a worse health state. Differences between time points were analysed using paired t-tests. ***p < 0.001, **p < 0.01, *p < 0.05. N = 600.

**S8 Table.** **Dyspnea-12 component scores at Weeks 0, 6 and 12.**

|  | Week 0 | Week 6 | Week 12 | Δ Week 0 to 6 | Δ Week 6 to 12 |
| --- | --- | --- | --- | --- | --- |
| I cannot get enough air | 0.8 (0.8) | 0.7 (0.8) | 0.5 (0.7) | -0.1 (-0.2, -0.1)*** | -0.2 (-0.2, -0.1)*** |
| I feel short of breath | 1.3 (0.8) | 1.1 (0.7) | 0.9 (0.7) | -0.2 (-0.2, -0.1)*** | -0.2 (-0.3, -0.2)*** |
| I have difficulty catching my breath | 1.0 (0.8) | 0.9 (0.8) | 0.7 (0.7) | -0.1 (-0.2, -0.1)*** | -0.2 (-0.3, -0.2)*** |
| My breath does not go all the way in | 0.9 (0.8) | 0.8 (0.8) | 0.7 (0.7) | -0.1 (-0.2, -0.1)*** | -0.1 (-0.2, -0.1)*** |
| My breathing is distressing | 0.6 (0.8) | 0.4 (0.6) | 0.3 (0.6) | -0.2 (-0.3, -0.2)*** | -0.1 (-0.1, 0.0)** |
| My breathing is exhausting | 0.7 (0.8) | 0.5 (0.7) | 0.5 (0.7) | -0.1 (-0.2, -0.1)*** | -0.1 (-0.1, 0.0)** |
| My breathing is irritating | 0.8 (0.9) | 0.6 (0.8) | 0.5 (0.7) | -0.2 (-0.3, -0.2)*** | -0.1 (-0.1, 0.0)** |
| My breathing is uncomfortable | 0.8 (0.8) | 0.7 (0.8) | 0.6 (0.7) | -0.2 (-0.2, -0.1)*** | -0.1 (-0.2, -0.1)*** |
| My breathing makes me agitated | 0.6 (0.8) | 0.4 (0.7) | 0.4 (0.7) | -0.2 (-0.2, -0.1)*** | -0.1 (-0.1, 0.0)* |
| My breathing makes me feel depressed | 0.6 (0.9) | 0.4 (0.7) | 0.4 (0.7) | -0.2 (-0.2, -0.1)*** | -0.1 (-0.1, 0.0)* |
| My breathing makes me feel miserable | 0.7 (0.9) | 0.5 (0.7) | 0.4 (0.7) | -0.2 (-0.3, -0.2)*** | -0.1 (-0.1, 0.0)*** |
| My breathing requires more work | 1.2 (0.8) | 1.0 (0.8) | 0.8 (0.7) | -0.2 (-0.2, -0.1)*** | -0.2 (-0.3, -0.1)*** |

Values for each timepoint are presented as mean (SD), while delta values are presented as mean (95% CI). Higher scores indicate a worse health state. Differences between time points were analysed using paired t-tests. ***p < 0.001, **p < 0.01, *p < 0.05. N = 525.

**S9 Table. Duke Activity Status Index component scores at Weeks 0, 6 and 12.**

|  | Week 0 | Week 6 | Week 12 | Δ Week 0 to 6 | Δ Week 6 to 12 |
| --- | --- | --- | --- | --- | --- |
| Can you take care of yourself (eating, dressing, bathing or using the toilet)? | No: 5  (1.0%)  Yes: 513 (99.0%) | No: 5  (1.0%)  Yes: 513 (99.0%) | No: 7  (1.4%)  Yes: 511 (98.6%) | No: 0  (0%)  Yes: 0  (0%) | No: 2  (0.4%)  Yes: -2  (-0.4%) |
| Can you walk indoors, such as around your house? | No: 1  (0.2%)  Yes: 517 (99.8%) | No: 0  (0%)  Yes: 518 (100%) | No: 1  (0.2%)  Yes: 517 (99.8%) | No: -1  (-0.2%)  Yes: 1  (0.2%) | No: 1  (0.2%)  Yes: -1  (-0.2%) |
| Can you walk a street or two on level ground? | No: 31  (6.0%)  Yes: 487 (94.0%) | No: 21  (4.1%)  Yes: 497 (95.9%) | No: 15  (2.9%)  Yes: 503 (97.1%) | No: -10  (-1.9%)  Yes: 10  (1.9%) | No: -6  (-1.2%)  Yes: 6  (1.2%) |
| Can you climb a flight of stairs or walk up a hill? | No: 57  (11.0%)  Yes: 461 (89.0%) | No: 53  (10.2%)  Yes: 465 (89.8%) | No: 43  (8.3%)  Yes: 475 (91.7%) | No: -4  (-0.8%)  Yes: 4  (0.8%) | No: -10  (-1.9%)  Yes: 10  (1.9%) |
| Can you run a short distance? | No: 445  (85.9%)  Yes: 73 (14.1%) | No: 407  (78.6%)  Yes: 111 (21.4%) | No: 351  (67.8%)  Yes: 167 (32.2%) | No: -38  (-7.3%)  Yes: 38  (7.3%)  *** | No: -56  (-10.8%)  Yes: 56  (10.8%)  *** |
| Can you do light work around the house, such as dusting or washing dishes? | No: 18  (3.5%)  Yes: 500 (96.5%) | No: 10  (1.9%)  Yes: 508 (98.1%) | No: 11  (2.1%)  Yes: 507 (97.9%) | No: -8  (-1.6%)  Yes: 8  (1.6%) | No: 1  (0.2%)  Yes: -1  (-0.2%) |
| Can you do moderate work around the house, such as hoovering, sweeping floors or carrying in groceries? | No: 170  (32.8%)  Yes: 348 (67.2%) | No: 108  (20.8%)  Yes: 410 (79.2%) | No: 77  (14.9%)  Yes: 441 (85.1%) | No: -62  (-12.0%)  Yes: 62  (12.0%)  *** | No: -31  (-5.9%)  Yes: 31  (5.9%)  *** |
| Can you do heavy work around the house, such as scrubbing floors, or lifting and moving heavy furniture? | No: 468  (90.3%)  Yes: 50 (9.7%) | No: 409  (79.0%)  Yes: 109 (21.0%) | No: 329  (63.5%)  Yes: 189 (36.5%) | No: -59  (-11.3%)  Yes: 59  (11.3%)  *** | No: -80  (-15.5%)  Yes: 80  (15.5%)  *** |
| Can you do gardening work, such as raking leaves, weeding or pushing a lawnmower? | No: 336  (64.9%)  Yes: 182 (35.1%) | No: 260  (50.2%)  Yes: 258 (49.8%) | No: 209  (40.3%)  Yes: 309 (59.7%) | No: -76  (-14.7%)  Yes: 76  (14.7%)  *** | No: -51  (-9.9%)  Yes: 51  (9.9%)  *** |
| Can you have sexual relations? | No: 223  (43.1%)  Yes: 295 (56.9%) | No: 160  (30.9%)  Yes: 358 (69.1%) | No: 129  (24.9%)  Yes: 389 (75.1%) | No: -63  (-12.2%)  Yes: 63  (12.2%)  *** | No: -31  (-6.0%)  Yes: 31  (6.0%)  *** |
| Can you participate in moderate recreational activities, such as golf, bowling, dancing or doubles tennis? | No: 434  (83.8%)  Yes: 84 (16.2%) | No: 374  (72.2%)  Yes: 144 (27.8%) | No: 300  (57.9%)  Yes: 218 (42.1%) | No: -60  (-11.6%)  Yes: 60  (11.6%)  *** | No: -74  (-14.3%)  Yes: 74  (14.3%)  *** |
| Can you participate in strenuous sports, such as swimming, singles tennis, football or skiing? | No: 501  (96.7%)  Yes: 17 (3.3%) | No: 469  (90.5%)  Yes: 49 (9.5%) | No: 414  (79.9%)  Yes: 104 (20.1%) | No: -32  (-6.2%)  Yes: 32  (6.2%)  *** | No: -55  (-10.6%)  Yes: 55  (10.6%)  *** |

Values are presented as count (%). Differences between time points were analysed using McNemar's chi-squared tests. ***p < 0.001, **p < 0.01, *p < 0.05. N = 518.

**S10 Table.** **EQ-5D-5L component scores at Weeks 0, 6 and 12.**

|  | Week 0 | Week 6 | Week 12 | Δ Week 0 to 6 | Δ Week 6 to 12 |
| --- | --- | --- | --- | --- | --- |
| Mobility | 2.1 (0.9) | 1.8 (0.8) | 1.8 (0.8) | -0.3 (-0.4, -0.2)*** | 0.0 (-0.1, 0.0) |
| Self-care | 1.4 (0.7) | 1.3 (0.6) | 1.2 (0.5) | -0.1 (-0.2, -0.1)*** | 0.0 (-0.1, 0.0) |
| Usual activities | 3.2 (1.0) | 2.7 (1.0) | 2.4 (1.0) | -0.6 (-0.6, -0.5)*** | -0.3 (-0.3, -0.2)*** |
| Pain / discomfort | 2.6 (0.9) | 2.4 (0.8) | 2.2 (0.9) | -0.3 (-0.3, -0.2)*** | -0.1 (-0.2, -0.1)*** |
| Anxiety / depression | 2.4 (0.9) | 2.0 (0.8) | 1.9 (0.8) | -0.3 (-0.4, -0.2) | -0.1 (-0.2, -0.1) |

Values for each timepoint are presented as mean (SD), while delta values are presented as mean (95% CI). Higher scores indicate a worse health state. Differences between time points were analysed using paired t-tests. ***p < 0.001, **p < 0.01, *p < 0.05. N = 526.

**S11 Table.** **World Health Organisation - Five Well-Being Index component scores at Weeks 0, 6 and 12.**

|  | Week 0 | Week 6 | Week 12 | Δ Week 0 to 6 | Δ Week 6 to 12 |
| --- | --- | --- | --- | --- | --- |
| I have felt cheerful and in good spirits | 2.1 (1.2) | 2.8 (1.2) | 3.1 (1.1) | 0.6 (0.5, 0.7)*** | 0.3 (0.2, 0.4)*** |
| I have felt calm and relaxed | 2.1 (1.2) | 2.7 (1.2) | 3.0 (1.2) | 0.6 (0.5, 0.7)*** | 0.3 (0.2, 0.4)*** |
| I have felt active and vigorous | 0.7 (0.9) | 1.5 (1.2) | 2.0 (1.4) | 0.8 (0.7, 0.9)*** | 0.4 (0.3, 0.5)*** |
| I woke up feeling fresh and rested | 0.8 (1.1) | 1.5 (1.3) | 1.8 (1.4) | 0.7 (0.5, 0.8)*** | 0.3 (0.2, 0.4)*** |
| My daily life has been filled with things that interest me | 1.7 (1.2) | 2.4 (1.3) | 2.7 (1.3) | 0.7 (0.6, 0.8)*** | 0.3 (0.2, 0.4)*** |

Values for each timepoint are presented as mean (SD), while delta values are presented as mean (95% CI). Lower scores indicate a worse health state. Differences between time points were analysed using paired t-tests. ***p < 0.001, **p < 0.01, *p < 0.05. N = 527.
